# Functional Analysis of the Tomato Immune Receptor Ve1 through Domain Swaps with Its Non-Functional Homolog Ve2

**DOI:** 10.1371/journal.pone.0088208

**Published:** 2014-02-05

**Authors:** Emilie F. Fradin, Zhao Zhang, Hanna Rovenich, Yin Song, Thomas W. H. Liebrand, Laura Masini, Grardy C. M. van den Berg, Matthieu H. A. J. Joosten, Bart P. H. J. Thomma

**Affiliations:** 1 Laboratory of Phytopathology, Wageningen University, Wageningen, The Netherlands; 2 Centre for BioSystems Genomics, Wageningen, The Netherlands; University of the West of England, United Kingdom

## Abstract

Resistance in tomato against race 1 strains of the fungal vascular wilt pathogens *Verticillium dahliae* and *V. albo-atrum* is mediated by the *Ve* locus. This locus comprises two closely linked inversely oriented genes, *Ve1* and *Ve2*, which encode cell surface receptors of the extracellular leucine-rich repeat receptor-like protein (eLRR-RLP) type. While Ve1 mediates *Verticillium* resistance through monitoring the presence of the recently identified *V. dahliae* Ave1 effector, no functionality for Ve2 has been demonstrated in tomato. Ve1 and Ve2 contain 37 eLRRs and share 84% amino acid identity, facilitating investigation of Ve protein functionality through domain swapping. In this study it is shown that Ve chimeras in which the first thirty eLRRs of Ve1 were replaced by those of Ve2 remain able to induce HR and activate *Verticillium* resistance, and that deletion of these thirty eLRRs from Ve1 resulted in loss of functionality. Also the region between eLRR30 and eLRR35 is required for Ve1-mediated resistance, and cannot be replaced by the region between eLRR30 and eLRR35 of Ve2. We furthermore show that the cytoplasmic tail of Ve1 is required for functionality, as truncation of this tail results in loss of functionality. Moreover, the C-terminus of Ve2 fails to activate immune signaling as chimeras containing the C-terminus of Ve2 do not provide *Verticillium* resistance. Furthermore, Ve1 was found to interact through its C-terminus with the eLRR-containing receptor-like kinase (eLRR-RLK) interactor SOBIR1 that was recently identified as an interactor of eLRR-RLP (immune) receptors. Intriguingly, also Ve2 was found to interact with SOBIR1.

## Introduction

Immunity in plants against pathogen attack is governed by immune receptors that detect appropriate ligands to activate defense. These ligands can either be microbial structures or ligands that occur as a consequence of plant-manipulating activities of microbial effectors [1], [2]. The host immune receptors activate various defence responses, often including a hypersensitive response (HR), which is necrosis of plant tissue surrounding the infection site that restricts further growth of the invading pathogen [3].

Verticillium wilt, caused by species of the soil borne fungal pathogen genus *Verticillium*, has been reported on over 200 dicotyledonous plant species [4], [5]. From tomato (*Solanum lycopersicum*) a locus providing *Verticillium* resistance has been cloned [6]. This *Ve* locus controls *V. dahliae* and *V. albo-atrum* strains belonging to race 1, while strains that are not controlled are assigned to race 2 [7]. The *Ve* locus is composed of two genes, *Ve1* and *Ve2*, that are highly homologous and that both encode extracellular leucine-rich repeat containing cell surface receptors of the receptor-like protein (eLRR-RLP) class [6], [8]. Ve1 and Ve2 are predicted to contain a signal peptide, an eLRR domain composed of two eLRR regions that are separated by a non-LRR island domain (also referred as C1, C3 and C2, respectively), a transmembrane domain, and a short cytoplasmic tail that lacks obvious signaling motifs besides putative homologs of mammalian endocytosis motifs [6]. Although Ve1 and Ve2 share 84% amino acid identity [6], only Ve1 mediates resistance against race 1 *Verticillium* strains in tomato [9]. However, it is presently unknown which domains of Ve1 are required to mediate resistance, and why Ve2 fails to provide resistance to race 1 *Verticillium* strains. For other eLRR-containing receptors, the eLRRs have been implicated in recognition specificity [10], [11], [12], [13], [14], [15], [16].

Several tomato eLRR-RLP-type immune receptors, referred to as Cf-proteins, which provide resistance against particular strains of the leaf mold fungus *Cladosporium fulvum* have been cloned [17], [18], [19], [20], [21], [22]. Through domain swaps and gene shuffling analyses, these Cf proteins were scrupulously dissected to identify specificity determining amino acids in their eLRR domains [16], [23], [24], [25], [26]. Overall, these studies demonstrated that specificity of the Cf proteins is determined by the number of eLRRs and specific amino acid residues that can either be clustered or scattered along the eLRR region. Furthermore, it was shown that specificity of the Cf proteins can be altered such that they are able to recognize other *C. fulvum* effectors.

Recently, through a population genomics approach in which we compared whole genome sequences of race 1 and race 2 strains, the effector of *Verticillium* race 1 strains that activate Ve1-mediated resistance was identified, designated Ave1. Transient expression of Ave1 by potato virus X (PVX) induced an HR in tomato carrying the *Ve1* gene [27]. Furthermore, simultaneous expression of *Ve1* and *Ave1* through *Agrobacterium tumefaciens*-mediated transient expression (agroinfiltration) in *Nicotiana tabacum* similarly induced an HR [27], [28]. Recently, it was demonstrated that functionality and specificity of tomato *Ve1* is maintained when it is expressed in Arabidopsis (*Arabidopsis thaliana*) plants, as *Ve1*-transgenic plants are resistant to race 1 strains of *V. dahliae* as well as *V. albo-atrum*, while race 2 strains remain virulent on these plants [9], [29]. Remarkably, however, *Ve1*-mediated resistance against *V. dahliae* does not seem to involve a hypersensitive response in Arabidopsis [30]. The use of Arabidopsis allows testing the functionality of chimeric Ve proteins in resistance against race 1 *Verticillium* strains. In this manuscript, we report on domain swaps between Ve1 and Ve2 that were expressed in *N. tabacum* and Arabidopsis to investigate functionality of the chimeric Ve proteins.

## Results

### Co-expression of Ave1 with HA-tagged Ve1 induces HR in tobacco

**Figure 1:**
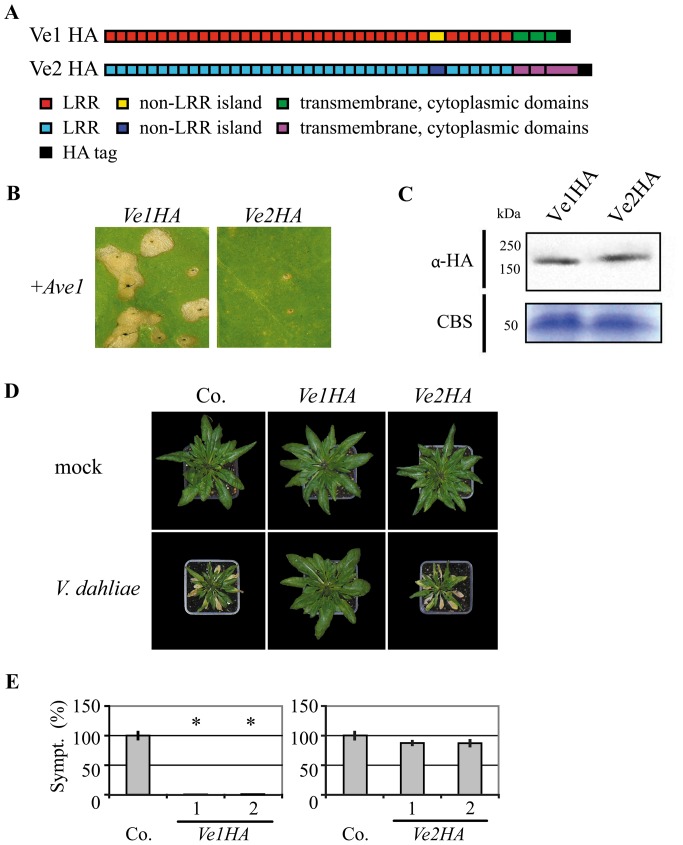
*Ve1*, but not of *Ve2*, provides resistance against *V. dahliae* race 1. (**A**) Schematic representation of the transgenically expressed Ve1 (Ve1HA) and Ve2 (Ve2HA) proteins. (**B**) Co-expression of *Ve1HA*, but not *Ve2HA*, with *Ave1* in tobacco results in a HR. Pictures were taken at five days post infiltration, and show representative leaves of at least 3 independent infiltrations. (**C**) HA-tagged Ve proteins were detected using HA antibody (α-HA). Coomassie-stained blots (CBS) showing the 50 kDa Rubisco band present in the input samples confirm equal loading. (**D**) Typical appearance of non-transgenic *sgs2* (Co.) and transgenic Arabidopsis *sgs2* lines that constitutively express *Ve1* or *Ve2* (*Ve1HA* and *Ve2HA,* respectively) upon mock-inoculation or inoculation with *V. dahliae* race 1. Photographs were taken at three weeks post inoculation and show a representative plant of the non-transgenic *sgs2* as well as a representative plant from one of the independent transgenic lines. (**E**) Quantification of Verticillium wilt symptoms (Sympt.) in Co. and transgenic lines. Bars represent quantification of symptom development shown as percentage of diseased rosette leaves with standard deviation. Co. is set to 100%. Asterisks indicate significant differences when compared with Co. (P<0.001). For each construct two independent transgenic lines are shown (1, 2).

To screen for functionality of constructs encoding domain swaps between Ve1 and Ve2, the coding sequence (CDS) of *V. dahliae Ave1* was cloned behind the *cauliflower mosaic virus* (CaMV) 35S promoter to generate expression construct Ave1. The CDSs of *Ve1* (FJ464556) and *Ve2* (FJ464558), fused to the CDS for an HA epitope tag, were cloned behind the CaMV 35S promoter to generate expression constructs Ve1HA and Ve2HA, respectively (Figure 1A). When tobacco leaves were co-infiltrated with a 1∶1 mixture of *A. tumefaciens* cultures carrying *Ave1* and *Ve1HA* respectively, HR was observed (Figure 1B). In contrast, co-expression of *Ave1* with *Ve2HA* in tobacco did not induce an HR (Figure 1B). Finally, stability of the HA-tagged Ve proteins was verified by immunoblotting (Figure 1C). For both Ve1-HA and Ve2-HA, the estimated size of the proteins based on comparison to the size markers exceeded the calculated sizes of the fusion proteins. However, similar discrepancies have previously been reported for other eLRR-containing cell surface receptors, such as CLV1 and Cf proteins, and have been attributed to N-glycosylation of the proteins [31], [32].

### *Ve1* provides resistance against *Verticillium* in *sgs2* plants

The Arabidopsis posttranscriptional gene silencing (PTGS) mutant *sgs2* [33], [34] typically shows little variation in transgene expression between individual transformants, and thus reduced numbers of transgenes need to be analysed [35]. Furthermore, we have previously demonstrated that the *sgs2* mutant displays enhanced *Verticillium* susceptibility when compared with wild type plants [36]. To assess the functionality of HA-tagged Ve proteins, *sgs2* plants were transformed with *Ve1HA* or *Ve2HA* and RT-PCR was performed to confirm expression of *Ve1* and *Ve2* in the transgenic lines (Figure S1). The resulting transgenic lines were subsequently challenged with the *V. dahliae* race 1 strain JR2. As expected, *Ve2HA-*expressing plants were as diseased as non-transgenic plants and displayed typical Verticillium wilt symptoms including stunting, wilting, anthocyanin accumulation, chlorosis, and necrosis (Figure 1D). In contrast, *Ve1HA-*expressing plants displayed clear *Verticillium* resistance as only few, if any, symptoms were observed on the inoculated plants (Figure 1D; 1E). These data show that HA-tagged Ve1 was able to provide *Verticillium* resistance, while HA-tagged Ve2 did not. Collectively, these results demonstrate that PTGS, which is affected in the *sgs2* mutant and is required for basal defence against *Verticillium* [36], is not required for Ve1-mediated resistance in Arabidopsis, and that HA-tagging of Ve1 does not affect its functionality.

### Ve1 and Ve2 comparison

**Figure 2:**
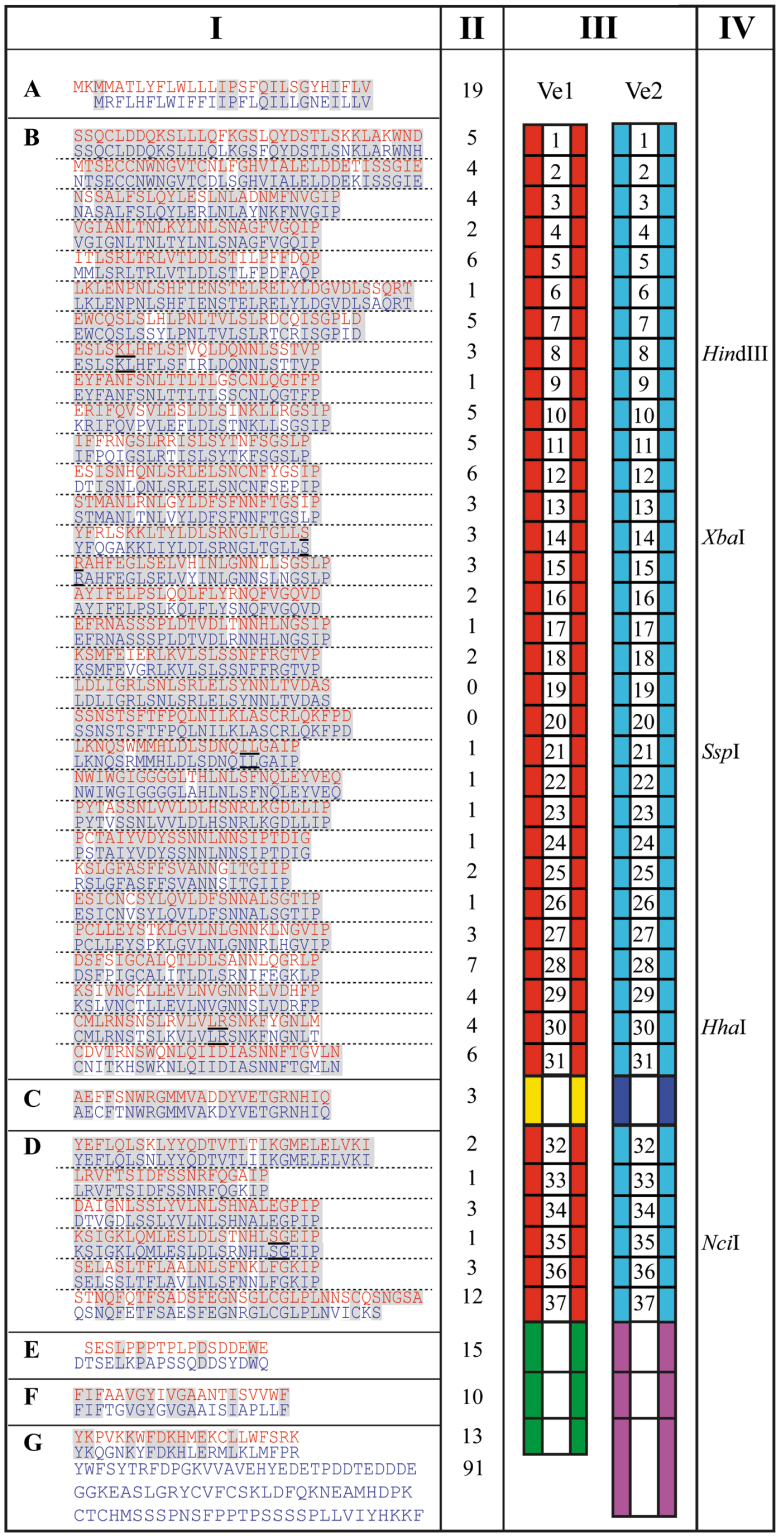
Protein sequence alignment of Ve1 and Ve2. Columns from Left to Right, I: Alignment of Ve1 (red) and Ve2 (blue) divided into: N-terminal signal peptide (A), leucine-rich repeat (eLRR) domains with each of the 37 eLRRs separated by a dashed line (B and D), non-LRR island domain (C), extracytoplasmic domain (E), transmembrane domain (F), and cytoplasmic domain (G). Conserved amino acid residues between Ve1 and Ve2 are highlighted. The underlined amino acid residues in eLRR8, eLRR14-15, eLRR21, eLRR30, and eLRR35 indicate positions that were used for domain swaps. II: Number of different amino acids between Ve1 and Ve2. III: Schematic representations of Ve1 and Ve2. Red and turquoise boxes represent the 37 eLRR domains of Ve1 and Ve2, respectively. Yellow and dark blue boxes represent the non-LRR island domains of Ve1 and Ve2, respectively. Green and mauve boxes represent the extracytoplasmic, transmembrane, and cytoplasmic domains of Ve1 and Ve2, respectively. IV: Restriction enzyme recognition site in eLRR8, eLRR14-15, eLRR21, eLRR30, and eLRR35 that were used for domain swaps.

Ve1 and Ve2 contain 37 imperfect eLRRs and share 84% amino acid identity (Figure 2). Of the 174 amino acid differences between Ve1 and Ve2, 117 are in the eLRRs and non-eLRR island domain. Furthermore, the Ve1 cytoplasmic tail is 91 amino acids shorter than the cytoplasmic tail of Ve2 (Figure 2). Remarkably, the region between eLRR19 and eLRR24 in the C1 domain is characterized by only a few amino acid differences. To identify regions that are required for Ve protein functionality, a domain swap strategy was designed, allowing the exchange of eLRRs between Ve1 and Ve2. The exact locations for the domain swaps between Ve1 and Ve2 were selected based on the presence of conserved endogenous restriction sites in the coding sequences of the two proteins (Figure 2).

### Chimeras containing the C-terminus of Ve2 do not provide *Verticillium* resistance

**Figure 3:**
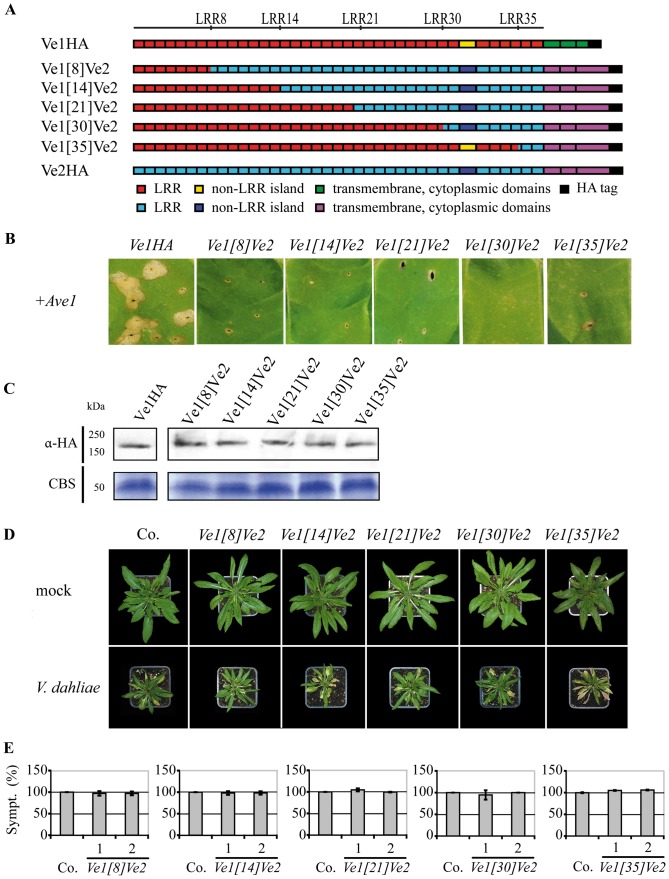
Functional characterization of Ve chimeric proteins that contain the C-terminus of Ve2. (**A**) Schematic representations of transgenically expressed Ve1 (Ve1HA) and Ve2 (Ve2HA) and the proteins encoded by the chimeric genes *Ve1* [*8*] *Ve2, Ve1* [*14*] *Ve2, Ve1* [*21*] *Ve2, Ve1* [*30*] *Ve2*, and *Ve1* [*35*] *Ve2.* The numbers indicate the eLRR at the site of the swap. (**B**) Chimeras containing the Ve2 C-terminus do not induce HR upon coinfiltration with *Ave1*. (**C**) Stability of chimeric Ve proteins is shown by immunoblotting using HA antibody (α-HA). Coomassie-stained blots (CBS) showing the 50 kDa Rubisco band present in the input samples confirm equal loading. (**D**) Typical appearance of non-transgenic *sgs2* (Co.) and transgenic Arabidopsis *sgs2* lines upon mock-inoculation or inoculation with *V. dahliae* race 1. Photographs were taken at three weeks post inoculation and show a representative plant of the non-transgenic *sgs2* as well as a representative plant from one of the independent transgenic lines. (**E**) Quantification of Verticillium wilt symptoms (Sympt.) in Co. and transgenic lines. Bars represent quantification of symptoms presented as percentage of diseased rosette leaves with standard deviation. Co. is set to 100%. No significant differences were monitored when compared with Co. (P<0.001). For each construct two independent transgenic lines are shown (1, 2).

To investigate whether Ve2 can be engineered to provide *Verticillium* resistance, we generated five chimeric Ve proteins; Ve1[8]Ve2, Ve1[14]Ve2, Ve1[21]Ve2, Ve1[30]Ve2, and Ve1[35]Ve2, in which the first 8, 14, 21, 30 or 35 eLRRs of Ve2 were replaced by those of Ve1, respectively (Figure 3A). Expression of none of the constructs resulted in HR upon co-expression with Ave1 by agroinfiltration in tobacco (Figure 3B). Stability of the chimeric Ve proteins was confirmed by immunoblotting (Figure 3C). To further investigate the functionality of the chimeric Ve proteins, Arabidopsis *sgs2* plants were transformed with the domain swap constructs and the transgenic lines were challenged with race 1 *V. dahliae*. RT-PCR analysis confirmed expression of the corresponding swap constructs (Figure S1). As expected, all transgenic lines were as diseased as wild type plants (Figure 3D–3E).

### eLRR30 to eLRR35 are required for Ve1 functionality

**Figure 4:**
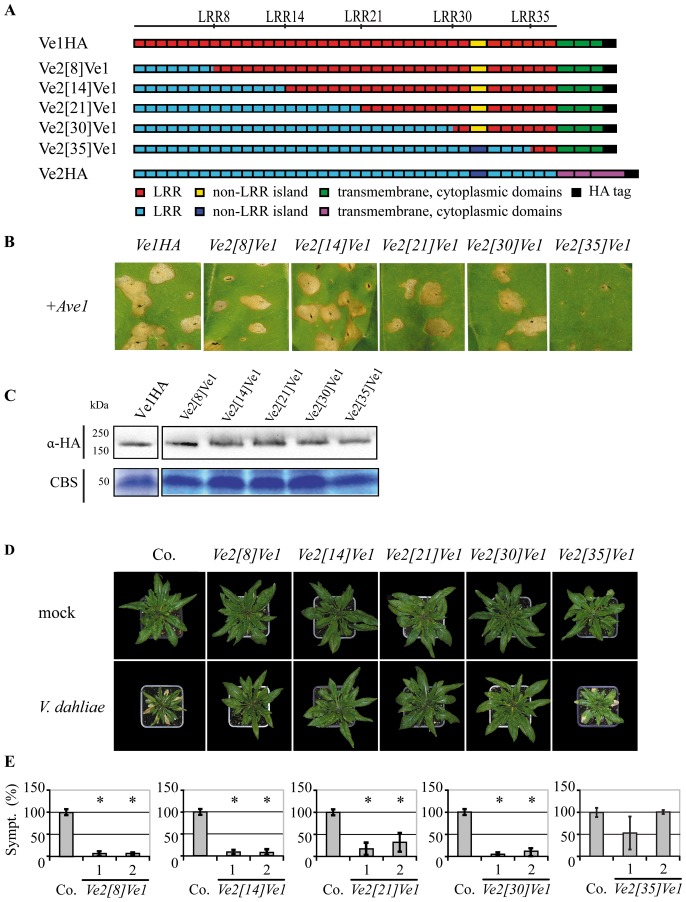
Functional characterization of Ve chimeric proteins that contain the C-terminus of Ve1. (**A**) Schematic representations of transgenically expressed Ve1 (Ve1HA) and Ve2 (Ve2HA) and the proteins encoded by the chimeric genes *Ve2* [*8*] *Ve1, Ve2* [*14*] *Ve1, Ve2* [*21*] *Ve1, Ve2* [*30*] *Ve1*, and *Ve2* [*35*] *Ve1.* The numbers indicate the eLRR at the site of the swap. (**B**) Typical appearance of tobacco leaves coinfiltrated with chimeric genes and *Ave1*. Pictures were taken at five days post infiltration, and show representative leaves for least 3 independent infiltrations. (**C**) Stability of chimeric Ve proteins is shown by immunoblotting using HA antibody (α-HA). Coomassie-stained blots (CBS) showing the 50 kDa Rubisco band present in the input samples confirm equal loading. (**D**) Typical appearance of non-transgenic sgs2 (Co.) and transgenic Arabidopsis *sgs2* lines upon mock-inoculation or inoculation with *V. dahliae* race 1. Photographs were taken at three weeks post inoculation and show a representative plant of the non-transgenic *sgs2* as well as a representative plant from one of the independent transgenic lines. (**E**) Quantification of Verticillium wilt symptoms (Sympt.) in Co. and transgenic lines. Bars represent quantification of symptoms presented as percentage of diseased rosette leaves with standard deviation. Co. is set to 100%. Asterisks indicate significant differences when compared with Co. (P<0.001). For each construct two independent transgenic lines are shown (1, 2).

To identify eLRRs that are required for Ve1 protein functionality, five Ve chimeric proteins were engineered; Ve2[8]Ve1, Ve2[14]Ve1, Ve2[21]Ve1, Ve2[30]Ve1, and Ve2[35]Ve1, in which the first 8, 14, 21, 30 or 35 eLRRs of Ve1 were replaced with those of Ve2, respectively (Figure 4A). Intriguingly, co-expression of Ave1 in combination with Ve2[8]Ve1, Ve2[14]Ve1, Ve2[21]Ve1, and Ve2[30]Ve1 resulted in HR in tobacco (Figure 4B). In contrast, tobacco leaves expressing the Ve chimera in which eLRR1 to eLRR35 of Ve1 were replaced with those of Ve2 did not show HR upon co-expression with Ave1 (Figure 4B). Again, stability of the chimeric Ve proteins was confirmed by immunoblotting (Figure 4C). To further investigate the chimeras, *sgs2* plants were transformed with *Ve2* [*8*] *Ve1, Ve2* [*14*] *Ve1, Ve2* [*21*] *Ve1, Ve2* [*30*] *Ve1* and *Ve2* [*35*] *Ve1*, and the resulting transgenic lines were challenged with race 1 *V. dahliae*. As expected based on the occurrence of HR in tobacco, expression of Ve2[8]Ve1, Ve2[14]Ve1, Ve2[21]Ve1 and Ve2[30]Ve1 in Arabidopsis resulted in *Verticillium* resistance, as the transgenes showed few to no symptoms (Figure 4D–4E). In contrast, plants carrying *Ve2* [*35*] *Ve1* displayed Verticillium wilt symptoms that were comparable to those of inoculated wild type plants (Figure 4D–4E). Collectively, these results suggest that the region between eLRR30 and eLRR35 is required for Ve1-mediated resistance, and that this region is not functional in Ve2.

**Figure 5:**
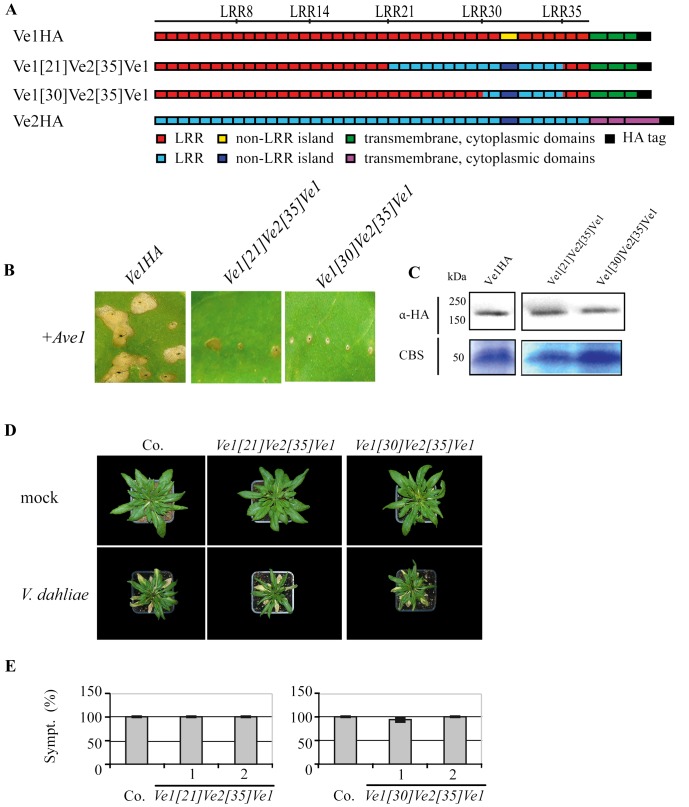
Analysis of the requirement of Ve1 eLRR30 to eLRR35 for mediating resistance against *V. dahliae* race 1. (**A**) Schematic representations of transgenically expressed Ve1 (Ve1HA) and Ve2 (Ve2HA) and the proteins encoded by the chimeric genes *Ve1* [*21*] *Ve2* [*35*] *Ve1* and *Ve1* [*30*] *Ve2* [*35*] *Ve1.* The numbers indicate the eLRR at the site of the swap. (**B**) Typical appearance of tobacco leaves coinfiltrated with chimeric genes and *Ave1*. Pictures were taken at five days post infiltration, and show representative leaves for least three independent co-infiltrations. (**C**) Stability of truncated and chimeric Ve proteins is shown by immunoblotting using HA antibody (α-HA). Coomassie-stained blots (CBS) showing the 50 kDa Rubisco band present in the input samples confirm equal loading. (**D**) Typical appearance of non-transgenic *sgs2* (Co.) and transgenic Arabidopsis *sgs2* lines upon mock-inoculation or inoculation with *V. dahliae* race 1. Photographs were taken at three weeks post inoculation and show a representative plant of the non-transgenic *sgs2* as well as a representative plant from one of the independent transgenic lines. (**E**) Quantification of Verticillium wilt symptoms (Sympt.) in Co. and transgenic lines. Bars represent quantification of symptoms presented as percentage of diseased rosette leaves with standard deviation. Co. is set to 100%. No significant differences were monitored when compared with Co. (P<0.001). For each construct two independent transgenic lines are shown (1, 2).

To further investigate the requirement of eLRR30 to eLRR35 for Ve1-mediated resistance, we generated Ve1[21]Ve2[35]Ve1 and Ve1[30]Ve2[35]Ve1, in which eLRR21 to eLRR35 and eLRR30 to eLRR35 of Ve1 were replaced with the corresponding eLRRs of Ve2, respectively (Figure 5A). Tobacco leaves expressing these Ve chimeras did not show HR upon co-expression with Ave1 (Figure 5B), while immunodetection confirmed stability of the chimeric proteins (Figure 5C). Arabidopsis plants expressing the constructs *Ve1* [*21*] *Ve2* [*35*] *Ve1* and *Ve1* [*30*] *Ve2* [*35*] *Ve1* displayed typical *Verticillium* wilt symptoms that were comparable to those of inoculated wild type plants and *Ve2* [*35*] *Ve1*-expressing plants (Figure 5D–5E). The expression of the corresponding constructs in the Arabidopsis transformants was verified by RT-PCR (Figure S1). Collectively, these results confirm that the region between eLRR30 and eLRR35 is required for Ve1-mediated resistance, and is not functional in Ve2.

### Deletion of eLRR1 to eLRR30 compromises Ve1 functionality

**Figure 6:**
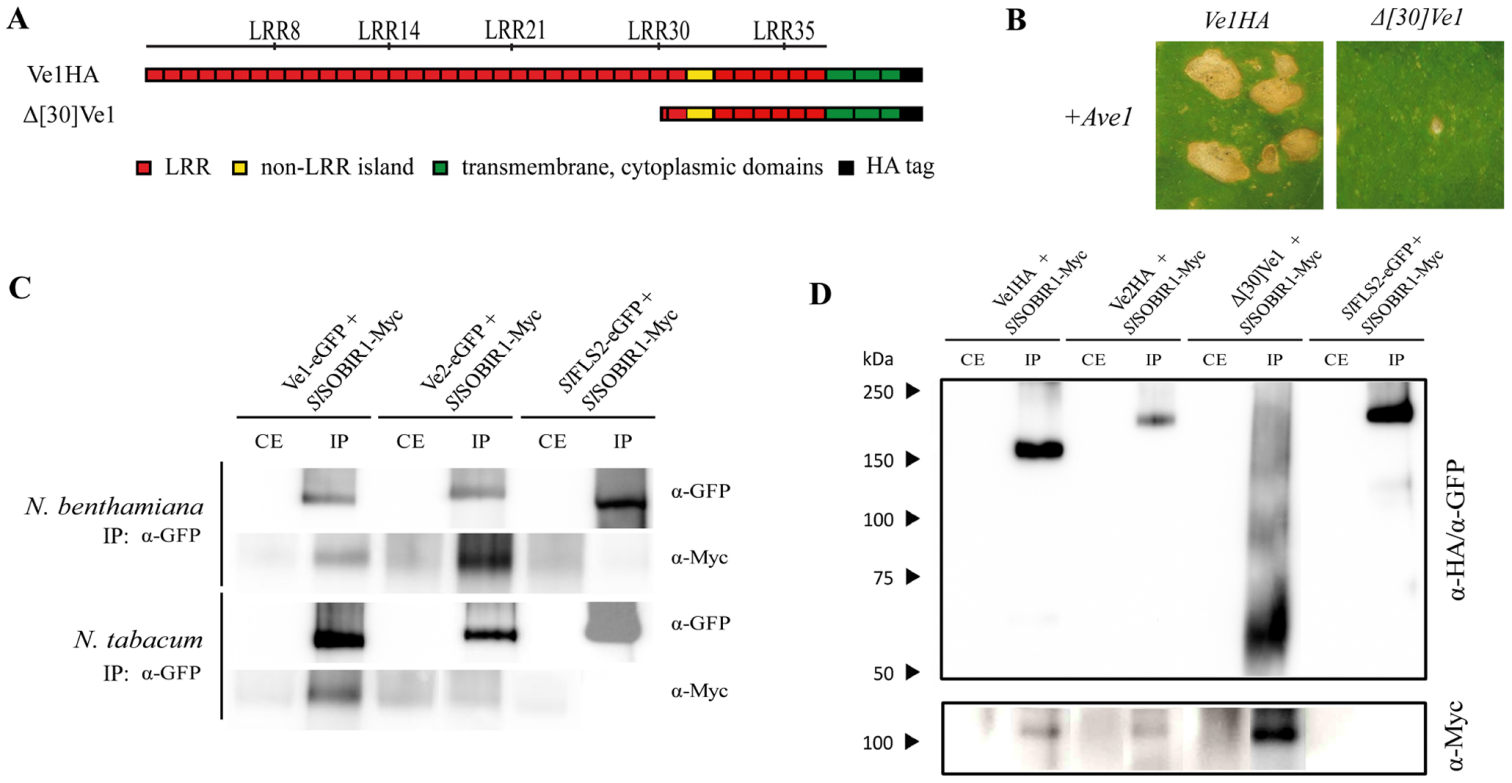
*In vivo* interaction of Ve proteins with the eLRR-RLK SOBIR1. (**A**) Schematic representations of transgenically expressed Ve1 (Ve1HA) and the truncated protein encoded by *Δ* [*30*] *Ve1*. (**B**) Typical appearance of tobacco leaves upon coexpression of Ave1 and Δ[30]Ve1. Pictures were taken at five days post infiltration, and show representative results for least three independent co-infiltrations. (**C**) Immunoprecipitation of protein extracts from *N. benthamiana* or *N. tabacum*. *Sl*SOBIR1-Myc is copurified with Ve1-eGFP and Ve2-eGFP upon immunoprecipitation with GFP-Trap beads (α-GFP). The eLRR-RLK *Sl*FLS2-eGFP that does not interact with *Sl*SOBIR1 is shown as a control [37]. (**D**) Upon immunoprecipitation of protein extracts from *N. tabacum* using α-HA affinity matrix and GFP-trap beads, *Sl*SOBIR1-Myc co-purifies with Ve1HA, Ve2HA and Δ[30]Ve1, whereas no signal is observed upon *Sl*FLS2-eGFP purification. IP: immunoprecipitation; CE: crude extract.

The observation that the region carrying eLRR1 to eLRR30 of Ve1 can be replaced by the corresponding region of Ve2 without compromising Ve1-mediated resistance suggests that this region is not required for Ve1 functionality or, alternatively, that this region is equally functional in both receptors. To investigate whether the region between eLRR1 and eLRR30 is required for Ve1 protein functionality, a truncated version of Ve1 was generated in which the first 30 eLRRs were deleted (Δ[30]Ve1; Figure 6A). Co-expression of Δ[30]Ve1 with Ave1 in tobacco did not induce HR (Figure 6B), while immunoblotting confirmed the stability of the truncated protein (Figure 6D). These data suggest that the region between eLRR1 and eLRR30 is indeed required for Ve1 protein functionality, and can be functionally replaced by the corresponding region of Ve2.

### The cytoplasmic tail is required for Ve1-mediated resistance

The finding that all chimeric Ve proteins that contain a Ve2 C-terminus are not functional suggests that the cytoplasmic tail is required for Ve1-mediated resistance. The C-terminus of Ve2 contains a PEST-like sequence that is found in proteins with short cytoplasmic half-lives and concludes with a KKX motif that may signal endoplasmic reticulum retention [6]. We recently demonstrated that GFP-tagged Ve1 localizes to the plasma membrane upon transient expression in tobacco epidermal cells [28]. To address the possibility that Ve2 is nonfunctional in mediating resistance to race 1 *Verticillium* strains due to differential localization when compared with Ve1, we compared their subcellular localization using green fluorescent protein (GFP) tagging. These data suggest that Ve1 and Ve2 share the same localization in tobacco epidermal cells (Figure S2).

**Figure 7:**
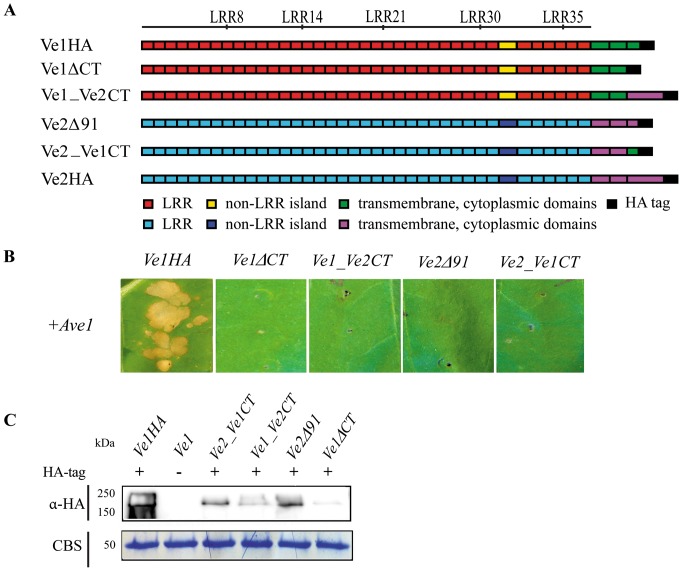
C-terminal cytoplasmic tail is required for Ve1-mediated resistance. (**A**) Schematic representations of transgenically expressed Ve1 (Ve1HA) and Ve2 (Ve2HA) and the proteins encoded by the truncated/chimeric genes *Ve1ΔCT, Ve1_Ve2CT, Ve2Δ91* and *Ve2_Ve1CT*. (**B**) C-terminal cytoplasmic tail is functional required for Ve1-mediated resistance, while Ve2 C-terminus is not functional. C-terminal truncated proteins and chimeras containing the Ve2 C-terminus do not induce HR upon coinfiltration with Ave1. Pictures were taken at five days post infiltration, and show representative leaves for least two independent co-infiltrations. (**C**) Stability of truncated or chimeric Ve proteins is shown by immunoblotting using HA antibody (α-HA). Coomassie-stained blots (CBS) showing the 50 kDa Rubisco band present in the input samples confirm equal loading.

To further investigate the role of cytoplasmic tail in Ve1-mediated resistance, we generated *Ve1ΔCT* and *Ve1_Ve2CT*, in which the coding sequence for the cytoplasmic tail of Ve1 was deleted or replaced by that of the cytoplasmic tail of Ve2, respectively (Figure 7A). Both Ve1ΔCT and Ve1_Ve2CT did not induce an HR when they were co-expressed with Ave1 in tobacco leaves (Figure 7B). These findings suggest that the cytoplasmic tail is required for Ve1-mediated resistance, and is not functional in Ve2.

The cytoplasmic tail of Ve2 is remarkably longer (91 amino acids) than the cytoplasmic tail of Ve1 (Figure 2). To investigate whether Ve2 can be engineered to activate immune signaling upon Ave1 perception by modulating its cytoplasmic tail, the cytoplasmic tail of Ve2 was truncated and replaced by the cytoplasmic tail of Ve1, resulting in constructs Ve2Δ91 and Ve2_Ve1CT, respectively (Figure 7A). However, tobacco leaves expressing either of these constructs did not develop HR upon co-expression with Ave1 (Figure 7B). These results indicate that non-functionality of Ve2 in providing race 1 *Verticillium* resistance cannot solely be attributed to its cytoplasmic tail and that other regions appear to be non-functional in Ve2 as well. Immunodetection confirmed stability of the diverse truncated and chimeric proteins (Figure 7C).

### Both Ve proteins interact with the receptor-like kinase SOBIR1

It was recently shown that the eLRR-RLK SOBIR1 constitutively interacts *in planta* with a broad range of eLRR-RLPs that act in development or in immunity, including Ve1 [37], [38], [39], [40]. In addition, SOBIR1 was found to be required for the Ve1-mediated hypersensitive response and immunity against *Verticillium* wilt in Arabidopsis and tomato [37]. Since SOBIR1 constitutively interacts with eLRR-RLPs that act either in development or in immunity, it was proposed that this protein functions as regulatory eLRR-RLK for eLRR-RLP-type of cell surface receptors [38]. To investigate whether perhaps absence of interaction of Ve2 with SOBIR1 could explain non-functionality of Ve2 in mediating race 1 *Verticillium* resistance, co-immunoprecipitations were performed to test the interaction of Ve1 and Ve2 with SOBIR1 both in *N. tabacum* and *N. benthamiana*. Interestingly, these assays revealed that Ve1 as well as Ve2 interacts with SOBIR1 (Figure 6C). Thus, it can be concluded that lack of Ve2 functionality cannot be attributed to the absence of interaction with the putative regulatory eLRR-RLK SOBIR1.

### eLRR1 to eLRR30 are not required for SOBIR1 interaction

Involvement of the eLRR domain in assembly of cell surface receptor complexes has recently been demonstrated [41], [42]. To investigate whether the region between eLRR1 and eLRR30 contributes to the interaction between Ve1 and SOBIR1, co-immunoprecipitations were performed using Δ[30]Ve1 and SOBIR1. Interestingly, these assays revealed that Δ[30]Ve1 still interacts with SOBIR1 (Figure 6D), suggesting that eLRR1 to eLRR30 of Ve1 do not contribute to the interaction with SOBIR1, and that this interaction is established through the C-terminus of the receptor.

## Discussion

In this manuscript we describe the analysis of a set of domain swaps between the eLRR-RLP-type cell surface receptor Ve1 and its close homolog Ve2. We show that the C-terminus and the region between eLRR30 to eLRR35 of Ve1 are crucial for resistance against *Verticillium* infection, and that these regions appear to be non-functional in Ve2. The finding that the first 30 eLRRs of Ve1 cannot be deleted without loss of Ve1 functionality suggests that the N-terminus is crucial for Ve1 function. Moreover, the observation that this region can be functionally replaced by the first 30 eLRRs of Ve2 suggests that this region is not impaired in Ve2.

All chimeric proteins in which eLRRs of Ve2 were replaced with those of Ve1 did not mediate HR upon co-expression of Ave1. Moreover, *sgs2* plants expressing Ve1[8]Ve2, Ve1[14]Ve2, Ve1[21] Ve2, Ve1[30]Ve2, and Ve1[35]Ve2, respectively, were susceptible towards *Verticillium*. These results show that the C-terminus of Ve2 is not functional. eLRR-RLPs typically have a short cytoplasmic tail of 20–30 amino acids lacking obvious signaling motifs, apart from motifs homologous to mammalian endocytosis motifs [8], [43]. The C-terminus of Ve2 is a rather a-typical cytoplasmic tail for an eLRR-RLP, as it is exceptionally long with 121 amino acids. In addition to the dileucine E/DXXXLφ and tyrosine YXXφ signal sequences that are thought to stimulate receptor-mediated endocytosis of mammalian receptors, the Ve2 C-terminus contains a PEST-like sequence that may induce protein degradation, and a KKF motif that has been suggested to promote endoplasmic reticulum retention [6]. However, the levels of expression of the chimeras and of the wild-type proteins shown by Western blot analysis seem to exclude the possible promotion of proteolysis. Ve1 only contains the dileucine E/DXXXLφ and tyrosine YXXφ sequences, although their functionality remains unclear. Although the Ve1 C-terminus lacks other signaling domains, it may interact with additional proteins which contribute to signal transduction. However, the recently identified regulatory eLRR-RLK SOBIR1, that broadly interacts with eLRR-RLP-type cell surface receptors, interacts with both Ve1 and Ve2, and therefore cannot explain the differential functionality of these proteins. Because SOBIR1 constitutively interacts with RLPs, irrespective of whether they act in immunity or in development, it has been suggested that SOBIR1 functions as a scaffold protein that stabilizes RLP-containing receptor complexes [37], [38]. The observation that *SOBIR1* silencing results in reduced immune receptor levels seems to support this hypothesis [37]. Our finding that the Ve2 receptor that is not functional in providing race 1 *Verticillium* resistance interacts with SOBIR1 may also suggest that SOBIR1 does not directly mediate Ve1-triggered immune signaling.

**Figure 8:**
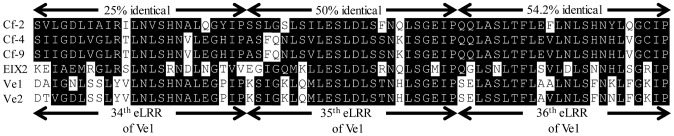
Sequence alignment of part of the C3 domain of selected tomato eLRR-RLP-type immune receptors. Identical and similar residues are indicated with black shading. The percentage of identical residues for each eLRR is indicated on top.

Intriguingly, the chimeras Ve2[8]Ve1, Ve2[14]Ve1, Ve2[21]Ve1, Ve2[30]Ve1 are able to trigger HR upon co-expression with Ave1 in tobacco. Furthermore, Arabidopsis *sgs2* plants expressing these chimeras were resistant against *Verticillium*, showing that the region containing the first 30 eLRRs of Ve2 is functional. This region includes the signal peptide (A-domain) and the major part of the C1 domain. The chimeric protein Ve2[35]Ve1, in which the first 35 eLRRs of Ve1 were replaced with those of Ve2, was not able to activate HR, and *Ve2* [*35*] *Ve1* transgenic *sgs2* remained susceptible towards *Verticillium*, suggesting that eLRRs 30 to 35 of Ve1 are required for *Verticillium* resistance, and are not functional in Ve2. This region includes two eLRRs from the C1 domain (eLRR30 and eLRR31), the island domain, and four eLRRs of the C3 domain (eLRR32 to eLRR35). Domain swap experiments between the eLRR-RLP receptor pairs Cf-4/Cf-9, Cf-2/Cf-5, Cf-9/Cf-9B demonstrated that ligand specificity is determined by the eLRR domain, specifically by the C1 domain [16], [23], [24], [25], [26]. So far, the role of the C3 domain remains unclear. However, a comparison of tomato RLPs Cf-2, Cf-4, Cf-9, EIX2, Ve1 and Ve2 shows that the C3 domain is more conserved (31.2% identical in amino acids) than the C1 domain (8.8% identical in amino acids). Moreover, in the C3 domain a number of highly conserved amino acids were observed, whereas the C3 domain of Cf-4 and Cf-9 is identical (Figure 8) [25]. Previous comparison of RLP sequences of Arabidopsis and rice has similarly shown that the C3 domains along with the extracytoplasmic and transmembrane domains are highly conserved [8], [44]. Domain-swaps between CLV2 and AtRLP38 (a CLV2-like RLP) demonstrated that the region from C3 to the C-terminus of AtRLP38 could substitute that of CLV2 without affecting CLV2 functionality [45]. The relatively high conservation of the C3 domain suggests that this region could be involved in interaction with co-receptors and other proteins that may form part of a receptor complex. The interaction of eLRR-containing cell surface receptors with other transmembrane receptors may be regulated by the transmembrane domain [46], [47] or even by the cytoplasmic domain [48]. Recent studies also revealed a crucial role for the eLRR domain as a platform for receptor interactions [41], [42]. Since we demonstrated that a truncated Ve1 protein that lacks the first 30 eLRRs still interacts with SOBIR1, we can hypothesize that this interaction is mediated by the C-terminus of the Ve1 protein, containing the remaining C3 domain the transmembrane domain and the cytoplasmic tail. In this light it is worthwhile to note that SOBIR1 only carries a short extracellular domain with only five eLRRs [49], [50].

In addition to C1 and C3 eLRRs, eLRR30 to eLRR35 encompass the non-LRR island domain (C2) which differs by three amino acids between Ve1 and Ve2. The C2 domain has been proposed to act as a flexible hinge region that facilitates the eLRR structure formation between the C1 and C3 regions [51]. However, requirement and function of the C2 domain has been shown to vary from one receptor protein to another [8], [43]. For instance, not all eLRR-RLPs and eLRR-RLKs contain a C2 domain [52]. Furthermore, the C2 domains of Cf-4 and Cf-9 are identical, suggesting that these regions are not involved in ligand specificity [16], [25]. Deletion of the C2 domain in CLV2 does not affect its functionality in plant development [45], whereas the C2 domains of BRI1 [53] and PSKR1 [54] are essential for functionality as they are directly involved in binding the ligands brassinolide and phytosulfokine, respectively [10], [55].

Several studies have addressed localization of Ve proteins based on GFP tagging, resulting in ambiguous results. It has been claimed that tomato Ve2 is localized in the endoplasmic reticulum [56], while the cotton Ve homolog GbVe was shown to be localized to the plasma membrane localization [57]. We recently demonstrated that GFP-tagged Ve1 localizes to the plasma membrane upon transient expression in tobacco epidermal cells [30]. In this manuscript we show that Ve1 and Ve2 are likely to share the same localization in tobacco epidermal cells (Figure S2). Nevertheless, localization of plasma membrane proteins based on GFP-tagging and over-expression should be taken with caution. It was previously shown that the epitope-tagged RLP Cf-9, when expressed under the control of the cauliflower mosaic virus 35S promoter, was found to localize at the plasma membrane [58], and at the ER [59]. It has been shown in various cases that overexpression of (membrane) proteins and epitope-tagging can result in mislocalization [60]. Indeed, when expressed under the control of Cf-9 cis-regulatory sequences in transgenic tobacco and using a Cf-9 specific antibody, Cf-9 was localized at the plasma membrane [60]. Overall, it is likely that immune receptors such as Ve1 localizes to the plasma membrane.

Overall, our results show that the C-terminus and the eLRR region from eLRR30 to eLRR35 are not functional in Ve2. However, the region carrying eLRR1 to eLRR30 is required for Ve1 functionality, and Ve chimeras in which eLRR1 to eLRR30 of Ve1 were replaced with those of Ve2 remained able to induce HR and provide resistance against race 1 *Verticillium*. Because for all known eLRR-RLPs the C1 domain determines ligand specificity, this may similarly be true for the Ve proteins. Thus, Ve2 may still interact with the Ave1 elicitor through the eLRRs of the C1 domain, but the C3 domain and the C-terminus of Ve2, which appear to be required for the interaction with co-receptors or downstream signaling components, may not be able to activate successful defense signaling. However, so far no direct interactions of Ve1 and Ve2 with the ligand Ave1 are shown. Presently, we cannot exclude the possibility that ligand perception is mediated by the island domain and/or LRRs 30 to 35 of Ve1, and Ve2 is completely unable to interact with Ave1. Future studies into the nature of the interaction of Ve1 with Ave1 will have to address this possibility.

## Materials and Methods

### Plant materials and manipulations

Plants were grown in soil in the greenhouse or in the climate chamber at 21°C/19°C during 16/8 hours day/night periods, respectively, with 70% relative humidity and 100 W·m^−2^ supplemental light when the light intensity dropped below 150 W·m^−2^. Arabidopsis transformations were performed as described previously [61] and single insertion T2 lines were selected by analyzing the segregation of glufosinateammonium resistance (Basta herbicide, Bayer CropScience). For each construct, at least two independent transgenic lines were used that showed no developmental aberrations. Inoculations with race 1 *V. dahliae* strain JR2 were performed as described previously [29]. For each non-transgenic *sgs2* and transgenic Arabidopsis *sgs2* lines, at least five plants were mock-inoculated and five plants were inoculated with *V dahliae* strain JR2. At three weeks post inoculation, photographs were taken and symptom development was assessed. To this end the percentage of diseased rosette leaves showing wilt and/or cholorosis was calculated. For each Arabidopsis line, susceptibility towards race 1 *V. dahliae* was investigated with at least three independent biological repeats, which yielded similar results. Statistical analysis was performed using Dunnett *t* test at *P* = 0.001.

### Generation C-terminal HA-tag fusions of Ve1 and Ve2

*pGEM-TdsVe1HA* was engineered to contain the tomato *Ve1* CDS (FJ464556) fused at the 3′ end to a CDS for the triple hemagglutinin (HA) epitope tag. To this end, the 392 bp fragment upstream of the *Ve1* stop codon was amplified from *P35S:Ve1* [9] with the Expand High-Fidelity PCR system enzyme mix (Roche) using primer pair *Ve1*SeqF6 and *Ve1HA*tagR (Table S1). The PCR fragment was cloned into *pGEM-T Easy* (Promega), sequenced using M13F and M13R (Table S1), and excised using *Nci*I and *Asc*I. In addition, construct *P35S:Ve1* was excised with *BamH*I and *Nci*I to obtain the first 2791 nucleotides of *Ve1*. Both fragments were cloned into *BamH*I*-* and *Asc*I*-*digested *pGEM-Tds* (a modified *pGEM-T Easy* vector that was engineered to contain a *Bam*HI and *Asc*I restriction site, Table S1), resulting in *pGEM-TdsVe1HA*. Similarly, *pGEM-TdsVe2HA* was engineered to encode tomato Ve2 (FJ464558) fused at the 3′ end to the triple HA tag. The 860 bp fragment upstream of the *Ve2* stop codon was amplified from *P35S:Ve2* [9] using primer pair *Ve2*SeqF6 and *Ve2HA*tagR (Table S1), cloned into *pGEM-T Easy*, sequenced, and excised with *Nci*I and *Asc*I. The first 2785 nucleotides of *Ve2* were excised from *P35S:Ve2* using *Bam*HI and *Nci*I. Subsequently, both fragments were cloned into *pGEM-Tds*, resulting in *pGEM-TdsVe2HA*.

For *in planta* expression of the Ve chimeras a variant of the Gateway vector *pB7WG2* [62] was engineered. To this end, the expression cassette between the restriction enzymes *Kpn*I and *Sac*I of *pB7WG2* was excised and replaced by the expression cassette present between the *Kpn*I and *Sac*I restriction sites of a binary vector pMOG800 variant [9], [63]. This resulted in the construct *pB7K40*, which contains the constitutive CaMV35S promoter, unique *Bam*HI and *Asc*I restriction sites, and the terminator of the potato proteinase inhibitor II (PiII) gene. Finally, the CDS encoding HA-tagged Ve1 and Ve2 were excised from *pGEM-TdsVe1HA* and *pGEM-TdsVe2HA*, respectively, and cloned into *BamH*I*-* and *Asc*I*-*digested *pB7K40*, resulting in *Ve1HA* and *Ve2HA,* respectively.

### Generation of constructs encoding Ve chimeras

The endogenous restriction sites *Hind*III, *Xba*I, *Ssp*I, *Hha*I, and *Nci*I that are conserved between *Ve1* and *Ve2* (Figure 2) were used to generate the domain-swaps. To generate the construct encoding a chimeric Ve protein that contains the first eight eLRRs of Ve1 and the remainder of the protein of Ve2 (pGVe1[8]Ve2), the *Ve1* fragment between *Bam*HI (in the multiple cloning site) and *Hind*III (conserved in the Ve proteins) was excised from *pGEM-TdsVe1HA* and cloned into *Bam*HI- and *Hind*III-digested *pGEM-TdsVe2HA*, resulting in *pGVe1* [*8*] *Ve2.* Similarly, to generate the construct encoding a chimeric Ve protein that contains the first 14 eLRRs of Ve1 and the remainder of the protein of Ve2 (pGVe1[14]Ve2), the *Ve1* fragment between *Bam*HI and *Xba*I was excised from *pGEM-TdsVe1HA* and cloned into *Bam*HI- and *Xba*I*-*digested *pGEM-TdsVe2HA.* To generate the construct encoding a chimeric Ve protein that contains the first 21 eLRRs of Ve1 and the remainder of the protein of Ve2 (pGVe1[21]Ve2), the *Ve1* and *Ve2* fragments between *Xba*I and *Ssp*I, and between *Ssp*I and *Asc*I, respectively, were excised from *pGEM-TdsVe1HA* and *pGEM-TdsVe2HA*, respectively. The excised fragments were then cloned into *XbaI*- and *Asc*I-digested *pGEM-TdsVe1HA.* To generate the construct encoding a chimeric Ve protein that contains the first 30 eLRRs of Ve1 and the remainder of the protein of Ve2 (pGVe1[30]Ve2), the *Ve1* and *Ve2* fragments between *Bam*HI and *Hha*I, and between *Hha*I and *Asc*I, respectively, were excised from *pGEM-TdsVe1HA* and *pGEM-TdsVe2HA.* The excised fragments were then cloned into *Bam*HI- and *Asc*I-digested *pGEM-Tds.* To generate the construct encoding a chimeric Ve protein that contains the first 35 eLRRs of Ve1 and the remainder of the protein of Ve2 (pGVe1[35]Ve2), the *Ve1* and *Ve2* fragments between *Bam*HI and *Nci*I, and between *Nci*I and *Asc*I, respectively, were excised from *pGEM-TdsVe1HA* and *pGEM-TdsVe2HA,* respectively. The excised fragments were then cloned into *Bam*HI- and *Asc*I-digested *pGEM-Tds.* Reciprocal constructs *pGVe2* [*8*] *Ve1, pGVe2* [*14*] *Ve1, pGVe2* [*30*] *Ve1,* and *pGVe2* [*35*] *Ve1*were generated following a similar cloning strategy as described above. For *pGVe2* [*21*] *Ve1*, the *Ve2* and *Ve1* fragments between *Bam*HI and *Ssp*I, and between *Ssp*I and *Asc*I, respectively, were excised from *pGEM-TdsVe2HA* and *pGEM-TdsVe1HA*, respectively. The excised fragments were then cloned into *Bam*HI- and *Asc*I-digested *pGEM-Tds.*

To generate *pGVe1* [*21*] *Ve2* [*35*] *Ve1,* a chimeric *Ve* CDS encoding LRR1 to LRR21 of Ve1, LRR21 to LRR35 of Ve2 and LRR35 to the C-terminus of Ve1, the chimeric fragment between *Ssp*I and *Asc*I was excised from *pGVe2* [*35*] *Ve1*, and the *Ve1* fragment between *Xba*I and *Ssp*I was excised from *pGEM-TdsVe1HA.* The excised fragments were cloned into *Xba*I- and *Asc*I-digested *pGEM-TdsVe1HA,* resulting in *pGVe1* [*21*] *Ve2* [*35*] *Ve1*. To generate *pGVe1* [*30*] *Ve2* [*35*] *Ve1*, a chimeric *Ve* CDS encoding LRR1 to LRR30 of Ve1, LRR30 to LRR35 of Ve2 and LRR35 to the C-terminus of Ve1, the chimeric fragment between *Hha*I and *Asc*I was excised from *pGVe2* [*35*] *Ve1,* and the *Ve1* fragment between *BamH*I and *Hha*I was excised from *pGEM-TdsVe1HA.* The excised fragments were then cloned into *BamH*I- and *Asc*I-digested *pGEM-Tds*, resulting in *pGVe1* [*30*] *Ve2* [*35*] *Ve1.*

Each domain-swap ligation was verified by sequencing (Table S1). Subsequently, all chimeras were excised from the *pGEM-Tds* vectors with *Bam*HI and *Asc*I and cloned into *Bam*HI- and *Asc*I-digested *pB7K40*, resulting in *Ve2* [*8*] *Ve1, Ve2* [*14*] *Ve1, Ve2* [*21*] *Ve1, Ve2* [*30*] *Ve1, Ve2* [*35*] *Ve1, Ve1* [*8*] *Ve2, Ve1* [*14*] *Ve2, Ve1* [*21*] *Ve2, Ve1* [*30*] *Ve2* and *Ve1* [*35*] *Ve2.*

To generate truncation constructs Ve1ΔCT and Ve2Δ91, the *Ve1* or *Ve2* coding sequence was PCR amplified using primers attB-Ve1-F and attB-Ve1ΔCT-R, or attB-Ve2-F and attB-Ve2Δ91-R, respectively (Table S1). The product was cloned into the pDONR207 vector according to manufacturer's instructions (Invitrogen, Carlsbad, California) to obtain entry vectors pDONR207::Ve1ΔCT and pDONR207::Ve2Δ91. The entry vectors were subsequently cloned into Gateway destination vector pGWB14 [64] using Gateway LR Clonase II enzyme mix (Invitrogen, Carlsbad, California) to generate expression construct Ve1ΔCT and Ve2Δ91 driven by the CaMV35S promoter.

To generate the construct encoding Ve1_Ve2CT, the *Ve1* fragment without the region encoding the cytoplasmic tail was PCR amplified using primers attB-Ve1-F and Ve1_Ve2CT-R, the region encoding the *Ve2* cytoplasmic tail was amplified using primers Ve2CT-F and attB-Ve2-R (Table S1). The PCR product encoding the Ve2 cytoplasmic tail was added to the Ve1 fragment that lacked the region encoding the cytoplasmic tail by overlap extension PCR. The product from the overlap extension PCR was cloned into the pDONR207 to obtain entry vector pDONR207::Ve1_Ve2CT. Similarly, the Ve2 coding sequence without cytoplasmic tail was PCR amplified using primers attB-Ve2-F and Ve2_Ve1CT-R. And the Ve1 cytoplasmic tail was amplified using primers Ve1CT-F and attB-Ve1-R (Table S1). The two PCR products were ligated by subsequent overlap extension PCR, and cloned into the pDONR207. Both pDONR207::Ve1_Ve2CT and pDONR207::Ve2_Ve1CT were subsequently cloned into Gateway destination vector pGWB14 to generate expression constructs Ve1_Ve2CT and Ve2_Ve1CT.

To generate truncation construct Δ[30]Ve1, the *Ve1* coding sequence was PCR amplified from *35S:Ve1* [9] using primers Δ[30]Ve1-F2 and C3R. A signal peptide sequence was added by subsequent PCR using primers SP-F and Ve1HAtagR. The product from the second PCR was cloned into the pENTR™/D TOPO vector according to manufacturer's instructions (Invitrogen, Carlsbad, California) to obtain entry vector pENTR:: Δ[30]Ve1. pENTR:: Δ[30]Ve1 was subsequently cloned into Gateway destination vector pSol2092 [28] using Gateway LR Clonase II enzyme mix (Invitrogen, Carlsbad, California) to generate expression construct Δ[30]Ve1 driven by the CaMV35S promoter.

### *A. tumefaciens*-mediated transient expression

The coding sequence of *V. dahliae Ave1* was cloned into Gateway destination vector pFAST_R02 [65] to generate an expression construct driven by the CaMV35S promoter. To generate Ve2 with a C-terminal GFP tag, the *Ve2* CDS was cloned into Gateway destination vector pSol2095 [28]. The expression constructs for GFP-tagged Ve1, *Sl*FLS2 and Myc-tagged SOBIR1 were described previously [28], [37]. The construct was transformed into *A. tumefaciens* strain GV3101 and infiltrated into tobacco plants (*N. tabacum* cv. Petite Havana SR1) as described previously [66]. Briefly, an overnight culture of *A. tumefaciens* cells was harvested at OD_600_ of 0.8 to 1 by centrifugation and resuspended to a final OD of 2. *A. tumefaciens* cultures containing constructs to express Ave1 and chimeric Ve protein were mixed in a 1∶1 ratio and infiltrated into leaves of five- to six-week-old tobacco plants. At five days post infiltration (dpi), leaves were examined for necrosis. Co-expression of Ave1 with Ve1 or functional chimeric Ve constructs triggered large necrotic spots at the injection sites. In contrast, no clear necrosis was observed at all in the infiltrated sector expressing *Ve2* or non-functional chimeric constructs. For every construct, the results were corroborated by at least three independent biological repeats in different tobacco plants.

### Protein extraction, co-immunoprecipitation and immunoblotting

For detection of HA-tagged Ve chimeras, *A. tumefaciens* containing the relevant expression constructs was infiltrated into tobacco plants as described previously [66]. Two days post infiltration, leaves were frozen in liquid nitrogen and ground to a fine powder. Proteins were dissolved in extraction buffer (150 mM Tris-HCL pH 7.5, 150 mM NaCl, 10 mM DTT, 10% glycerol, 10 mM EDTA, 0.5% polyvinylpyrrolidon [PVPP], 1% IGEPAL CA-630 [NP-40] and 1% protease inhibitor cocktail [Roche]). Samples were then centrifuged at 4°C for 15 min at 5000 g and the supernatant was passed through a 0.45 µm filter. The immunopurifications and immunoblotting were done as described previously [67].

For the co-immunoprecipitation of *Sl*SOBIR1-Myc with the different Ve fusion proteins, constructs were agroinfiltrated in a 1∶1 ratio into tobacco plants. Infiltrated leaves were harvested after one day and ground to a fine powder. The protein extraction, immunopurifications and immunoblotting were performed as described previously [67]. All experiments have been repeated at least twice.

### RNA isolation and Reverse Transcription-PCR

Two-week-old Arabidopsis seedlings were collected and total RNA was isolated using the Qiagen RNAeasy Kit (Qiagen, Valencia, California). First-strand cDNA was synthesized from 1 µg of total RNA, using the SuperScript™ III cDNA synthesis kit (Invitrogen, Carlsbad, California) according to the manufacturers' instructions. RT-PCR was conducted with primers Ve-RT-F and Ve-RT-R (Table S1) in a total volume of 25 µl with 17.9 µl water, 5 µl 5x PCR buffer, 0.5 µl dNTPs, 0.5 µl of each primer, 0.1 µl GoTaq polymerase (Promega, Madison, Wisconsin) and 1 µl of first-strand cDNA. The primer pairs AtRubisco-F3 and AtRubisco-R3 (Table S1) were used to amplify the Arabidopsis RuBisCo gene as endogenous loading control. PCR reactions were performed for 30 cycles, denaturing at 95°C for 30s, annealing at 55°C for 30s, and elongation at 72°C for 30s. The generated PCR products were evaluated by agarose gel electrophoresis.

### Confocal microscopy

The plasma membrane marker, mCherry-HVR [68]), was co-infiltrated with the Ve-GFP fusions into leaves of 6-week-old tobacco plants (*N. tabacum* cv. Petite Havana SR1). The fluorescence was imaged at 24 hours after infiltration using a Carl Zeiss LSM 710 confocal laser scanning microscopy system.

## Supporting Information

Figure S1 **Expression of** ***Ve1*** **,** ***Ve2*** **and** ***Ve*** **chimeras in transgenic Arabidopsis.** As an endogenous control, a fragment of the Arabidopsis RuBisCo gene was amplified from cDNA. For each construct two transgenic lines are shown (1, 2).
(TIF)

Figure S2 **Subcellular localization of GFP-tagged Ve1 and Ve2 in in epidermal cells of** ***N. tabacum*** **leaves.** The plasma membrane marker, mCherry-HVR, was transiently co-expressed with the GFP fusions. The fluorescence was imaged at 24 hours after infiltration. From left to right: GFP fluorescence, fluorescence of the plasma membrane marker mCherry-HVR, differential interference contrast (DIC), and a merged image. Bar  =  20 µm.
(TIF)

Table S1 **Primers used in this study.**
(DOCX)
