# Supplementary figures and images for: Functional Analysis of the Tomato Immune Receptor Ve1 through Domain Swaps with Its Non-Functional Homolog Ve2
Source: PLoS One. 2014 Feb 5;9(2):e88208. doi: 10.1371/journal.pone.0088208 (PMC3914901; doi:10.1371/journal.pone.0088208)

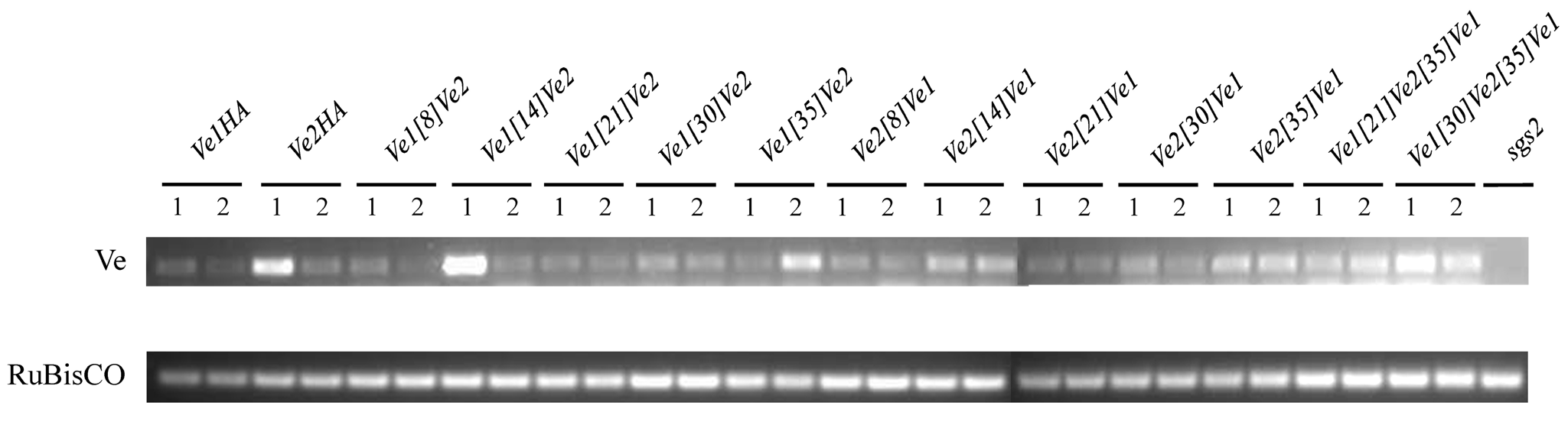

Supplement: Figure S1 — Expression of Ve1 , Ve2 and Ve chimeras in transgenic Arabidopsis. As an endogenous control, a fragment of the Arabidopsis RuBisCo gene was amplified from cDNA. For each construct two transgenic lines are shown (1, 2). (TIF) [file pone.0088208.s001.tif]

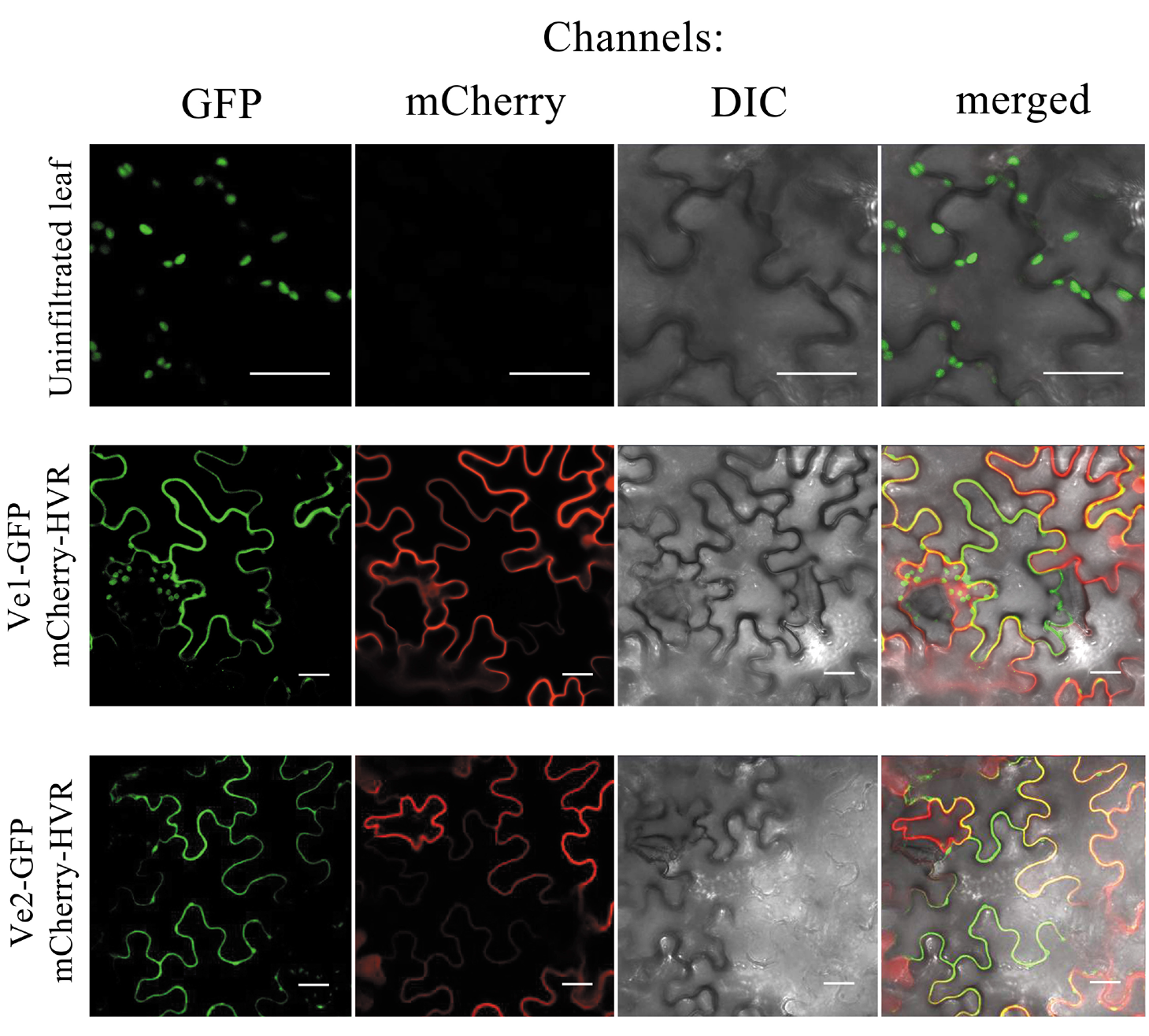

Supplement: Figure S2 — Subcellular localization of GFP-tagged Ve1 and Ve2 in in epidermal cells of N. tabacum leaves. The plasma membrane marker, mCherry-HVR, was transiently co-expressed with the GFP fusions. The fluorescence was imaged at 24 hours after infiltration. From left to right: GFP fluorescence, fluorescence of the plasma membrane marker mCherry-HVR, differential interference contrast (DIC), and a merged image. Bar = 20 µm. (TIF) [file pone.0088208.s002.tif]
