# Supplementary material for: Functional Analysis of the Tomato Immune Receptor Ve1 through Domain Swaps with Its Non-Functional Homolog Ve2
Source: PLoS One. 2014 Feb 5;9(2):e88208. doi: 10.1371/journal.pone.0088208 (PMC3914901; doi:10.1371/journal.pone.0088208)
Supplement: Table S1 — Primers used in this study. (DOCX) [file pone.0088208.s003.docx]

**Table S1.** Primers used in this study.

| **Primer name** | **Sequence (5’-3’)^a^** | **Description^b^** |
| --- | --- | --- |
| Ve1SeqF6 | acctgtcaacaaaccacctgtcc | *Ve1* 3x*HA* tag (C) |
| Ve1HAtagR | **ggcgcgcc**tcaagcgtaatctggaacatcgtatgggtaagcgtaatctggaacatcgtatgggtaagcgtaatctggaacatcgtatgggtactttcttgaaaaccaaagc | *Ve1* 3x *HA* tag (***Asc*I**) (C) |
| Ve2SeqF6 | taatcatcaaaggcatggagctg | *Ve2* 3x *HA* tag (C) |
| Ve2HAtagR | **ggcgcgcc**tcaagcgtaatctggaacatcgtatgggtaagcgtaatctggaacatcgtatgggtaagcgtaatctggaacatcgtatgggtaaaactttttgtgatatatg | *Ve2* 3x *HA* tag (***Asc*I**) (C) |
| attB-Ve1-F | GGGGACAAGTTTGTACAAAAAAGCAGGCTATGAAAATGATGGCAACTCT | *Ve* chimera (C) |
| attB-Ve1ΔCT-R | GGGGACCACTTTGTACAAGAAAGCTGGGT AAACCACACAACTGAAATAG | *Ve* chimera (C) |
| AttB-Ve2-F | GGGGACAAGTTTGTACAAAAAAGCAGGCTATGAGATTTTTACACTTTCTATG | *Ve* chimera (C) |
| attB-Ve2Δ91-R | GGGGACCACTTTGTACAAGAAAGCTGGGT TCTAGGAAACATCAGTTTAAG | *Ve* chimera (C) |
| Ve1_Ve2CT-R | CAAAGTATTTGTTTCCTTGCTTGTAAAACCACACAACTGAAATAG | *Ve* chimera (C) |
| Ve2CT-F | gcaaatactatttcagttgtgtggtttTACAAGCAAGGAAACAAA | *Ve* chimera (C) |
| Ve2_Ve1CT-R | CAAACCATTTCTTCACTGGCTTGTAAAACAACAGAGGTGCAA | *Ve* chimera (C) |
| Ve1CT-F | gcaatctccattgcacctctgttgtttTACAAGCCAGTGAAGAAA | *Ve* chimera (C) |
| attB-Ve1-R | GGGGACCACTTTGTACAAGAAAGCTGGGTACTTTCTTGAAAACCAAAG | *Ve* chimera (C) |
| attB-Ve2-R | GGGGACCACTTTGTACAAGAAAGCTGGGTAAAACTTTTTGTGATATATGACT | *Ve* chimera (C) |
| Δ[30]Ve1-F2 | TCCAATAAATTCTATGGA | *Ve* chimera (C) |
| SP-F | ATGAAAATGATGGCAACTCTGTACTTCCTATGGCTTCTCTTGATTCCCTCGTTTCAAATCTTATCAGGATACCACATTTTCTTGGTTTCCAATAAATTCTATGGA | *Ve* chimera (C) |
| C3R | TCACTTTCTTGAAAACCAAAGCAAGCATTTC | *Ve* chimera (C) |
| M13F | cgccagggttttcccagtcacgac | pGEM-T (S) |
| M13R | tcacacaggaaacagctatgac | pGEM-T (S) |
| *Ve1*SeqF3 | ggaacaatttactcagcgggagc | *Ve1* chimera (S) |
| *Ve1*SeqR3 | tcttggacagtcgaaaatatggg | *Ve1* chimera (S) |
| *Ve1*SeqR4 | ccatgactgattcttgagatcgg | *Ve1* chimera (S) |
| *Ve1*SeqR5 | ccttgtaagttattcgcactga | *Ve1* chimera (S) |
| *Ve1*SeqR6 | caagggcattgtgtgacagattc | *Ve1* chimera (S) |
| *Ve1*R | **ggcgcgcc**tcactttcttgaaaacgaaagc | *Ve1* chimera (***Asc*I**) (S) |
| *Ve2*SeqR3 | aaggttcgaaatggtgtctggta | *Ve2* chimera (S) |
| *Ve2*SeqR4 | ccttaagcctcccaacttcaaac | *Ve2* chimera (S) |
| *Ve2*SeqR5 | gctaccgagaaaaaggaggca | *Ve2* chimera (S) |
| *Ve2*SeqR6 | tgcgtcctgtctccacgtaatc | *Ve2* chimera (S) |
| *Ve*2SeqR7 | ttggtgctggtttcaactctga | *Ve2* chimera (S) |
| Ve-RT-F | CATATTGAAATTAGCGTCTTGTCGG | *Ve* chimera (RT) |
| Ve-RT-R | ACCGAGAAAAAGGAGGCAAAAC | *Ve* chimera (RT) |
| AtRubisco-F3 | gcaagtgttgggttcaaagctggtg | Arabidopsis Rubisco (RT) |
| AtRubisco-R3 | ccaggttgaggagttactcggaatgctg | Arabidopsis Rubisco (RT) |

^a^ Restriction site in bold

^b^The type of experiment for which the primers were used is indicated in brackets (C: cloning, RT: real-time PCR or semi-quantitative RT-PCR, S: sequencing)
